# Cyclin H predicts the poor prognosis and promotes the proliferation of ovarian cancer

**DOI:** 10.1186/s12935-020-01406-5

**Published:** 2020-07-16

**Authors:** Chen Peng, Yansong Yang, Li Ji, Panpan Yang, Xiaoqing Yang, Yuquan Zhang

**Affiliations:** 1 Department of Gynecology and Obstetrics, Affiliated Hospital of Nantong University, No. 20 Xisi Road, Nantong, 226001 Jiangsu China; 2 Department of Radiology, Affiliated Cancer Hospital of Nantong University, No. 48 Qingnianxi Road, Nantong, 226001 China; 3 Clinical Medicine of Nantong University, No. 9 Seyuan Road, Nantong, 226001 China

**Keywords:** Cyclin H, CDK2, Ovarian cancer, Cell cycle

## Abstract

**Background:**

Cell cycle dysregulation plays a key role in the pathogenesis of malignant tumors. As a part of the CDK-activating kinase (CAK) trimeric complex, cyclin H is necessary to regulate the cell cycle and proliferation. This investigation aims to characterize the clinical significance and the biological functions of cyclin H in ovarian cancer.

**Methods:**

Immunohistochemical staining was performed on 60 ovarian cancer cases, and a correlation between cyclin H expression and the clinical characteristics of ovarian cancer was analyzed. The function of cyclin H in ovarian cancer was further explored using HO8910 cells and a subcutaneous xenograft model of nude mice.

**Result:**

Cyclin H was slightly expressed in grade 1 ovarian cancer but highly expressed in grade 2 and grade 3 cancerous tissues. The Spearman’s rank correlation analysis showed that the expression of cyclin H is positively correlated with the tumor grade, the FIGO stage, histological grade, and the peritoneal metastasis of ovarian cancer and is also positively correlated with the Ki67 and p-CDK2 in ovarian cancer. Additionally, we found that the five-year survival rate was higher in patients expressing low cyclin H than those expressing high cyclin H. Further, knockdown of cyclin H was achieved using an shRNA in HO8910 ovarian cancer cell line. Silencing cyclin H resulted in a G1/S cell cycle arrest in ovarian cancer cells suppressing its growth. The Ki67 expression was also decreased in cyclin H silenced ovarian cancer.

**Conclusion:**

These results suggest that high expression of cyclin H predicts the poor prognosis and promotes the growth of ovarian cancer by regulating the cell cycle.

## Background

Ovarian cancer is the seventh most common tumor in women and also the most lethal gynecological malignancy with a high recurrence and mortality rate [1–3]. A five-year survival rate of an ovarian cancer patient is less than 35% [4]. Most ovarian cancer patients do not get diagnosed until the advanced stages because they do not show symptoms in the early stages [3, 5, 6]. Therefore, investigating the pathogenesis of ovarian cancer and finding new target proteins that are closely related to the occurrence, development, and prognosis of ovarian cancer may increase the early diagnosis rate and provide new ideas in treating the disease.

Deregulation of cell cycle and abnormal signaling pathways caused by various reasons are general features of the tumor cells [7]. Excessive cell proliferation, blocked differentiation, and impaired apoptosis play a key role in the occurrence and development of tumors. Clarifying the molecular mechanism of cell cycle regulation in tumor cells may provide the basis for developing tumor treatment strategies [8, 9]. The three main types of molecules involved in cell cycle regulation are as follows: cyclin, cyclin-dependent kinase (CDK), and cyclin-dependent kinase inhibitor (CDKI). The expression of cell cycle regulators is linked to tumor development, prognosis, and treatment response [10]. Cyclin H, a member of the cyclin family, together with CDK7 and MAT1 forms the CDK-activating kinase (CAK) trimeric complex, which is necessary for the regulation of cell cycle and proliferation. Abnormal expression of cyclin H is reported in a variety of tumors such as breast cancer [11], esophageal cancer [12], endometrial cancer [13], and gastrointestinal stromal tumors [14]. However, the clinical significance and the biological function of cyclin H in ovarian cancer remains unclear.

Immunohistochemical staining was used in this study to evaluate the expression of cyclin H in 60 pathological specimens of ovarian cancer with different grades. The correlation of cyclin H with the clinical characteristics, prognosis, and the proliferation parameters of tumor patients were analyzed. Additionally, we down-regulated the expression of cyclin H in ovarian cancer cells using shRNA and explored its effect on cell cycle and tumor growth. This study revealed that cyclin H is highly expressed in high-grade ovarian cancers and promotes the growth of ovarian cancer by regulating the cell cycle.

## Methods

### Pathological samples

Paraffin embedded ovarian cancer specimens were obtained from 60 patients who underwent surgery in the Department of Obstetrics and Gynecology at Affiliated Hospital of Nantong University from June 2009 to April 2013. All the tumors were from newly diagnosed ovarian cancer patients and also from the patients who did not receive any treatment before surgery. Among these, four patients’ survival information was missing. All the investigations in this study were performed after obtaining informed consent. The study was approved by the Research Ethics Committee of the Affiliated Hospital of Nantong University.

### Immunohistochemistry (IHC) and quantification

Formalin-fixed and paraffin-embedded sections were prepared for all the tissues and reviewed by three pathologists. The protein expression of cyclin H, Ki67, and p-CDK2 in ovarian cancer was determined by IHC. In short, ovarian cancer tissue sections were first dewaxed in xylene, then rehydrated through gradient ethanol, and heated in Tris–EDTA buffer (pH 8.0) for 15 min in a microwave oven to retrieve the antigen. After cooling it at room temperature and rinsing with phosphate-buffered saline, slides were incubated with 3% hydrogen peroxide for 10 min to block endogenous peroxidase activity. Next, it was incubated with 2% bovine serum albumin (Beyotime Biotechnology, Shanghai, China) for 2 h at room temperature to block nonspecific reactions. Tissue sections were then incubated with cyclin H monoclonal antibody (1:50, MA5–32331, Thermo Fisher Scientific, Waltham, MA, USA), Ki67 monoclonal antibody (1:300, ab92742, Abcam, Cambridge, MA, USA) or p-CDK2 polyclonal antibody (1:100, PA5–38128, Thermo Fisher Scientific) at 4 °C overnight. All the slides were finally visualized using the Envision kit (Dako, Glostrup, Denmark) as per the instructions and then counterstained with hematoxylin, dehydrated, and sealed with coverslips. The sections were imaged under an optical microscope and evaluated in a blinded manner. More than 500 cells in each slide were counted to determine the mean positive percentage and the expression range was scored as follows: 0 (< 20%), 1 (20% to 50%), 2 (51% to 75%) and 3 (> 75% points). For the frozen sections of tumors from nude mice, dewaxing steps were omitted.

### Cell culture and transfection

Human ovarian carcinoma HO8910 cell line was used in this study, which was obtained from the Cell Bank of the Chinese Academy of Sciences (Shanghai, China). The HO8910 cell line was cultured in RPMI 1640 (Gibco, Grand Island, NY) supplemented with 10% fetal bovine serum (FBS, Gibco), 2 mM l-glutamine, 0.11 g/L Sodium Pyruvate, 100 U/mL penicillin–streptomycin mixture (Gibco) at 37 °C in 5% CO_2_ incubator. Cyclin H shRNA(sequence: 5′-CCG GCG ACC TGG TAG AAT CTC TCT ACT CGA GTA GAG AGA TTC TAC CAG GTC GTT TTT G-3′) was synthesized by Sangon Biotech (Shanghai, China), cloned into a pLenti-CMV-puro vector and then transfected to HO8910 cells using FuGENE transfection reagent (Promega, Madison, WI, USA). The stable transfected cell lines were obtained after screening the cells with 1 µg/mL of puromycin. Empty plasmids were transfected into HO8910 cells and used as a control.

### Cell viability assay

Cells were seeded in a 96 well plate (3000 cells per well) and cultured in complete RPMI 1640 medium (with 10% fetal bovine serum). Cell viability was measured at time points 0, 12, 24, 48, and 72 h after seeding by cell counting kit-8 (C0038, Beyotime, Shanghai, China) according to the manufacturer’s instructions. Five wells were used for each time point, and their cell viability was measured and compared to the initial cell viability.

### Western blot

HO8910 cells were harvested by trypsin, washed with PBS, and collected after centrifugation. Cell pellets were resuspended with cell lysate buffer and incubated on ice for 20 min. For tumor samples, 1 mL of cell lysate buffer was added to 0.1 g of tissue followed by homogenization and centrifugation. Prepared samples were run on SDS-PAGE gel and transferred to polyvinylidene fluoride membranes (0.2 µm, Millipore, USA). After blocking with 5% non-fat milk, the membranes were incubated with cyclin H antibody (1:1500, MA5–32331, Thermo Fisher Scientific), MAT1 antibody (1:1000, ab129176, Abcam), CDK7 antibody (1:1000, 2916, Cell Signaling Technology, Danvers, MA, USA), p-CDK2 antibody (1:100, PA5–38128, Thermo Fisher Scientific) or CDK2 antibody (1:1000, 2546, Cell Signaling Technology) and then incubated with HRP goat anti-rabbit or mouse IgG (ABclonal, Wuhan, China). Membranes were finally visualized using an enhanced chemiluminescence reagent (34095, Thermo Pierce) and GAPDH (1:2000, Abcam) was used as a control.

### Quantitative PCR

Total RNA of HO8910 cells was isolated using Trizol (Invitrogen) and reverse transcription was done using a commercial kit (RR047B, TaKaRa, Tokyo, Japan) that contained gDNA Eraser to eliminate genomic DNA contamination. The relative mRNA level of cyclin H was determined by real-time quantitative PCR (RR420L, TaKaRa), where β-actin was used as a control. The following primers were used in the reaction: cyclin H forward, 5′-TGT TCG GTG TTT AAG CCA GCA-3′; cyclin H reverse, 5′-TCC TGG GGT GAT ATT CCA TTA CT-3′; β-actin forward, 5′-TCG AGC ACG GCA TCG TCA CCA-3′; β-actin reverse, 5′-ATA GCA ACG TAC ATG GCT-3′.

### Cell cycle detection

HO8910 cells were synchronized by deprivation of serum 4q 72 h. The serum-free medium was then replaced by complete medium, and cells were cultured for another 48 h before collection. Cells were harvested using trypsinization, and a single-cell suspension was prepared before fixing and staining them. Propidium staining was done using a detection kit (C1052, Beyotime) and was detected by BD FACSCanto II (San Diego, CA, USA). The data was analyzed using Modfit software, and the percentage of cells in each phase were counted.

### Tumor model

Female BALBC/c nude mice (5–6 weeks old, 14–16 g) were purchased from Shanghai SLAC Laboratory Animal Co., Ltd. (Shanghai, China) and maintained in specific pathogen-free conditions. HO8910 cells (5 × 10^5^/mouse) in 100 µL of PBS were inoculated subcutaneously to the nude mice, in the flank. Tumor diameters were measured with digital calipers every other day, and the tumor volume was calculated by the following formula: Volume = (width)^2^ × length/2. All animal experiments were performed according to the Guidelines for the Care and Use of Laboratory Animals (No. 55 issued by Ministry of Health, China on January 25, 1998) and were approved by the Ethics Committee for Laboratory Animals of Nantong University.

### Statistical analysis

IBM SPSS was used for all statistical analyses. Chi square test was used for clinicopathological categorical variables, and the overall survival was analyzed by the Kaplan–Meier method. *T* test was used for studying the difference between the two groups. Spearman’s rank correlation analysis was used to measure the association between the expression of cyclin H, Ki67, and p-CDK2. Differences were considered statistically significant at *P *< 0.05.

## Result

### High expression of cyclin H in ovarian cancer

**Fig. 1:**
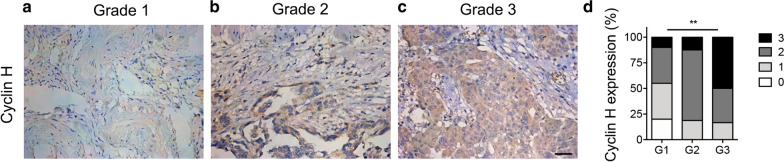
Immunohistochemistry staining of Cyclin H in ovarian cancer tissues. The expression of Cyclin H in ovarian cancer of grade 1 (**a**), grade 2 (**b**), and grade 3 (**c**) was measured by immunohistochemistry staining, and representative images are displayed. Scale bar, 20 µm. **d** Quantification of Cyclin H expression in ovarian cancer (P < 0.001)

To investigate the expression and the potential role of cyclin H in ovarian cancer, immunohistochemical staining was performed on 60 cases of ovarian cancer, and cyclin H expression was scored subsequently. Representative results of immunohistochemistry are shown in Fig. 1. In the ovarian cancer tissues, cyclin H is localized in the nucleus and the cytoplasm. The mean percentage of cyclin H positive cells was 33.16% (ranged from 3.10% to 77.48%). Cyclin H was slightly expressed in grade 1 ovarian cancer while highly expressed in high grade 2 and grade 3 samples (Fig. 1a–c). High cyclin H expression was significantly correlated with the increased tumor grade (Fig. 1d and Table 1).

**Table 1:** Relationship between clinicopathological characteristics and Cyclin H protein expression in ovarian cancer

Clinicopathological characteristic	No. Cases	Cyclin H Expression				R	P
		0	1	2	3		
Age (years)							
< Mean	27	1	8	10	8	− 0.012	0.925
≥ Mean	33	3	6	16	8		
FIGO stage, n (%)							
I	15	3	5	6	1	0.389	0.002^*^
II	6	0	3	1	2		
III	31	1	5	16	9		
IV	8	0	1	3	4		
Histologic subtype, n (%)							
Serous papillary adenocarcinoma	42	2	9	17	14	− 0.228	0.079
Endometrioid adenocarcinoma	7	1	2	2	2		
Clear cell carcinoma	3	0	1	2	0		
Mucinous carcinoma	8	1	2	5	0		
Histologic grade, n (%)							
G1	20	4	7	7	2	0.466	0.0017^***^
G2	16	0	3	11	2		
G3	24	0	4	8	12		
Residual tumor (cm), n (%)							
≤ 1	45	3	12	20	10	0.166	0.194
> 1	15	1	2	6	6		
Chemotherapy, n (%)							
Platinum only	1	0	1	0	0	0.146	0.263
Other/platinum	40	2	7	20	11		
None	10	2	6	6	5		
Malignant tumor cells in peritoneal fluid							
Present	31	3	10	13	5	0.308	0.016*
Absent or rare	29	1	4	13	11		

Statistical analyses were performed by Pearson χ^2^ test

* P < 0.05, **P < 0.01

### Correlation between cyclin H expression and Clinicopathological Variables of ovarian cancer

Further, to explore the potential physiological or the pathological role of cyclin H in ovarian cancer, the correlation between cyclin H and the clinicopathological parameters was analyzed and summarized in Table 1. The result of spearman Spearman’s rank correlation analysis showed that cyclin H in ovarian cancer was not associated with age, histological subtype, residual tumor size, and chemotherapy. Interestingly, Cyclin H positively correlated with the FIGO (International Federation of Gynecology and Obstetrics) stage (P = 0.002), histologic grade (P = 0.0017), and the peritoneal metastasis. Compared to the patients with no peritoneal metastasis, cyclin H expression was significantly increased in patients with malignant tumor cells in peritoneal fluid (P = 0.016). These data suggest that the abnormal expression of cyclin H is closely related to the pathological characteristics of ovarian cancer.

### Cyclin H positively correlates with Ki67 and p-CDK2 in ovarian cancer

**Fig. 2:**
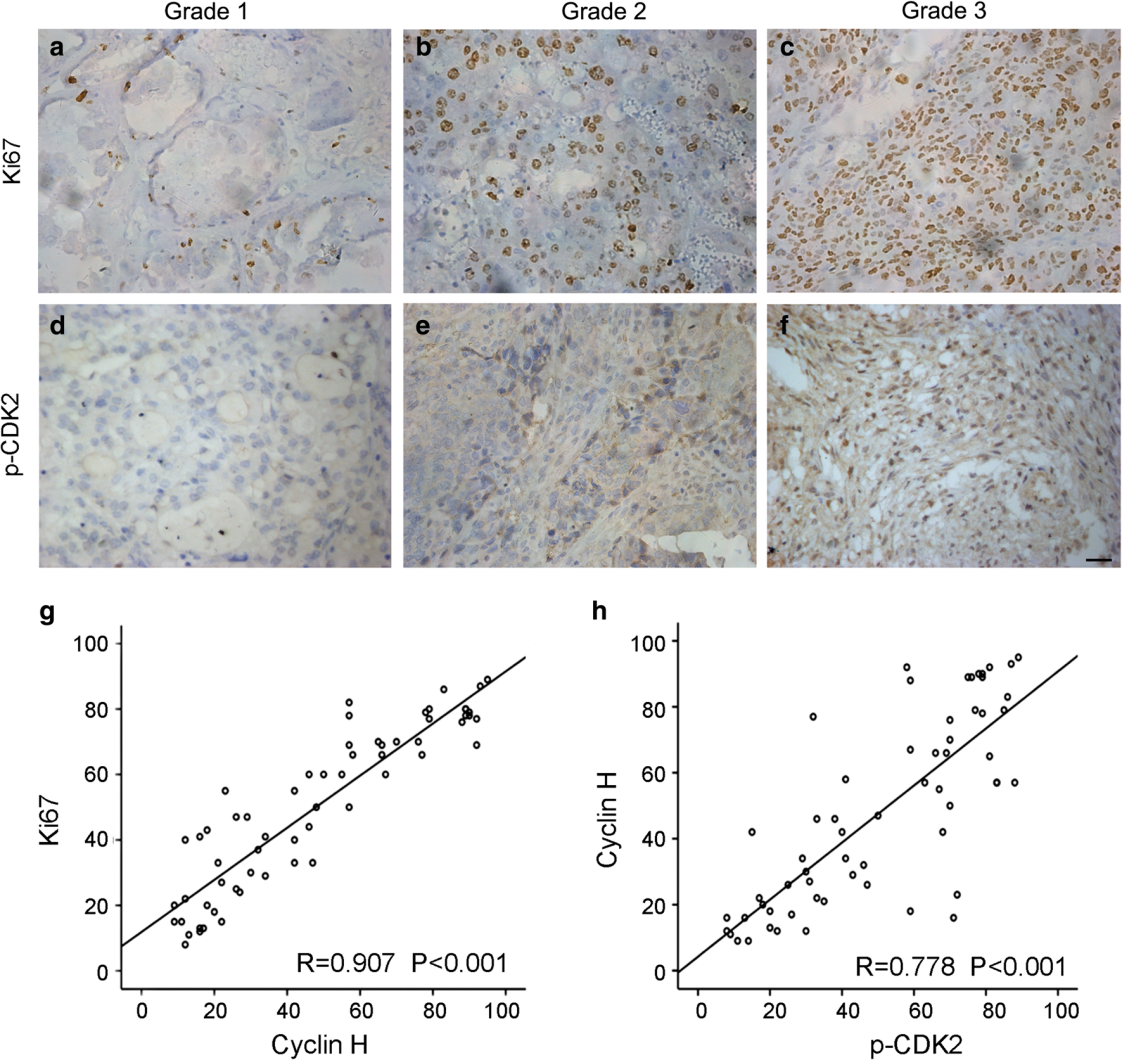
Cyclin H correlates with Ki67 and p-CDK2 in ovarian cancer. **a**–**c** Immunohistochemistry staining of ki67 in ovarian cancer of grade 1 (**a**), grade 2 (**b**), and grade 3 (**c**). **d–f** Immunohistochemistry staining of p-CDK2 in ovarian cancer. Scale bar, 20 µm. **g** Correlation between the expression of Ki67 and Cyclin H in ovarian cancer. **h** Cyclin H was positively correlated with p-CDK2 in ovarian cancer

Cyclin H is a well-known component of CDK-activating kinase, which contributes to the cell cycle by mediating CDK activation [15]. Therefore, the expression of cell cycle-related molecules, Ki67, and p-CDK2 were determined by immunohistochemical staining. As expected, Ki67 was highly expressed in grade 2 and grade 3 ovarian cancer when compared to the grade 1 specimens (Fig. 2a–c). The expression trend of p-CDK2 (Fig. 2d–f) was similar to that of Ki67 and cyclin H. Additionally, the results showed that Cyclin H was positively correlated to Ki-67 (r = 0.907; P < 0.001) (Fig. 2g) and p-CDK2 (r = 0.788; P < 0.001) (Fig. 2h) expression in 60 ovarian cancer tissues, demonstrating the involvement of cyclin H in cell cycle regulation of ovarian cancer.

### Cyclin H expression is associated with poor patient outcome in ovarian cancer

**Fig. 3:**
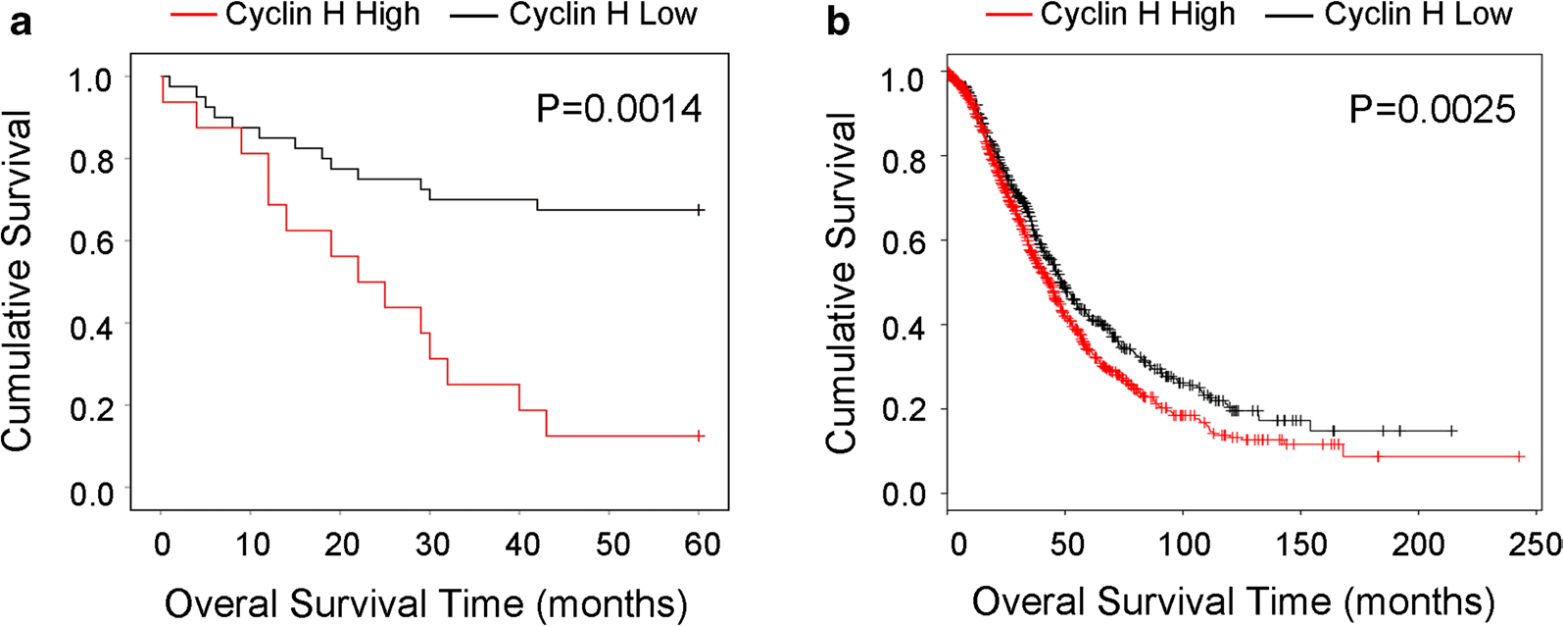
Survival analysis in ovarian cancer patients. **a** Analysis of patients showing a low level of cyclin H (score ≤ 2, n = 40) and those with high expression of cyclin H (score > 2, n = 16). **b** Analysis of 1656 ovarian cancer patients showed that high expression of cyclin H predicted poor prognosis. The Kaplan–Meier plots were generated by Kaplan–Meier Plotter (http://www.kmplot.com)

To determine whether the expression of cyclin H predicts the survival of ovarian cancer patients or not, we analyzed the relationship of cyclin H protein and the mRNA expression with the survival of ovarian cancer patients. Carcinoma specimens were divided into cyclin H low expressers (score ≤ 2, n = 40) and cyclin H high expressers (score > 2, n = 16) according to the immunohistochemical staining results. Moreover, according to Kaplan–Meier analysis, the five-year survival rate of patients was significantly higher in the cyclin H low-expression group compared to that in the cyclin H high-expression group (P = 0.0014) (Fig. 3a). Additionally, the relationship between cyclin H mRNA expression level and the survival of ovarian cancer patients was analyzed using publicly available datasets (n = 1656, http://www.kmplot.com) according to the instructions. Patients with low mRNA expression of cyclin H showed a significant improvement in survival time (Fig. 1a). These data demonstrate that high expression of cyclin H correlates with the poor prognosis of ovarian cancer.

### The knockdown of cyclin H results in the G1/S cell cycle arrest of ovarian cancer cells

**Fig. 4:**
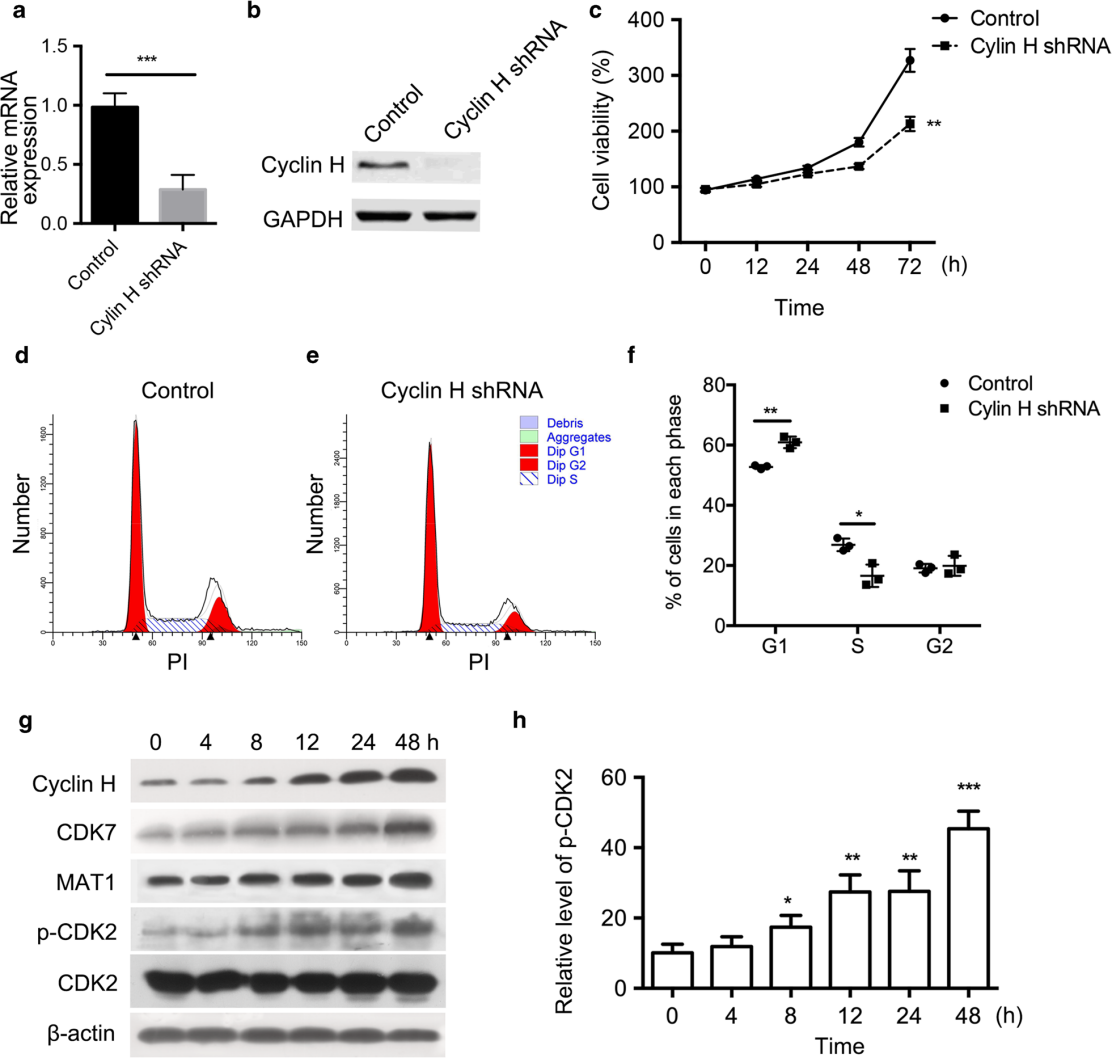
Cyclin H regulates the proliferation and cell cycle of ovarian cancer cells. **a** mRNA level of cyclin H in vector and cyclin H shRNA transfected ovarian cancer HO8910 cells. **b** The protein expression of cyclin H was detected by Western blot. **c** Proliferation difference between control and cyclin H silencing HO8910 cells. PI staining was performed to measure the percentage of cells in each cell cycle phase using Modfit software (**d** and **e**). **f** Quantification of the percentage cells in each cell cycle phase after transfection with cyclin H shRNA. **g** Expression of cyclin H, CDK7, MAT1, p-CDK2, and CDK2 in HO8910 cells after serum deprivation and refeeding. Serum-starved HO8910 cells were cultured in serum-containing medium for 4, 8, 12, 24, and 48 h, and cell lysates were analyzed by western blot. **h** Relative level of p-CDK2 was normalized to the total level of CDK2 at each time point. * P < 0.05, **P < 0.01, ***P < 0.001

To clarify the role of cyclin H in the proliferation and cell cycle of ovarian cancer cells, HO8910 cells were used because of its high level of cyclin H (Additional file 1: Fig. S1). The expression of cyclin H was knocked down by shRNA in HO8910 cells, and its efficiency was determined by quantitative PCR (Fig. 4a) and western blot (Fig. 4b). The cyclin H level was greatly reduced in cyclin H shRNA transfected cells compared to the control group. Cell viability and cell cycle of HO8910 cells were measured by CCK8 and flow cytometry, respectively. Cyclin H silenced HO8910 cells showed weaker proliferative capacity, which was indicated by lower cell viability than that of the control group cells (Fig. 4 C), while the migration and invasion were not affected (Additional file 2: Fig. S2). G1/S cell cycle arrest was observed in cyclin H interfered cells, where the percentage of cells in the G1 phase increased while the percentage of cells in S phase decreased compared to those in the control group (Fig. 4d–f). The expression of cyclin H, MAT1, CDK7, and p-CDK2 in HO8910 cells gradually increased after serum stimulation (Fig. 4g, h). These results suggest that cyclin H is a positive regulator of ovarian cancer cell proliferation.

### Cyclin H regulates the growth of ovarian cancer

**Fig. 5:**
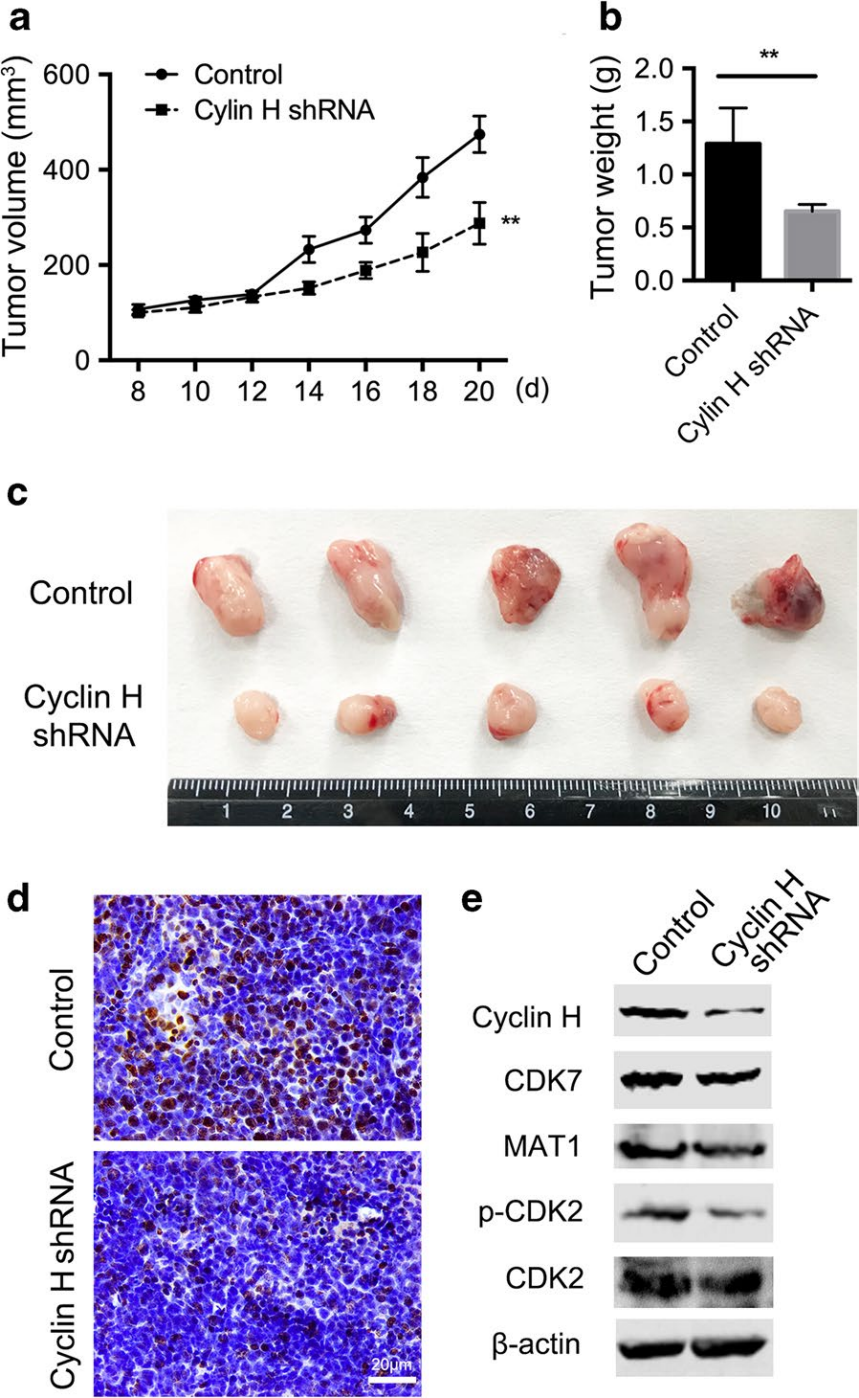
Cyclin H promotes the growth of ovarian cancer. Nude mice were implanted with cyclin H silenced HO8910 cells or control cells, and tumor volume was monitored regularly (**a**). **b** Tumor weight was measured at day 20 after mice were sacrificed. **c** Tumors from control or cyclin H shRNA group. Data are mean ± SEM. Representative results of one of three independent experiments. **P < 0.01. **d** Immunohistochemical staining immunohistochemical staining of Ki67 in tumors from nude mice. Tumor tissues were isolated and fixed for the frozen section, and the thickness was 30 µm. Bar = 20 µm. (E) Expression of cyclin H, CDK7, MAT1, p-CDK2, and CDK2 in xenografts

We confirmed the role of cyclin H in ovarian cancer in vivo using a subcutaneous nude mouse tumor model. Cyclin H shRNA or the control shRNA transfected HO8910 cells were implanted subcutaneously to the mice, and the development of tumors was monitored every other day. Tumors were observed on day 8, and their volume was determined. Mice were sacrificed, the tumor was removed and weighed on day 20. Tumor’s weight and size in the cyclin H shRNA group was much lighter and smaller, respectively than those in the control group, indicating that cyclin H knockdown significantly reduced ovarian cancer growth (Fig. 5a–c). Though, no obvious change in body weight and daily behavior was observed between the two groups. IHC staining shows that Ki67 decreased in cyclin H silenced tumors (Fig. 5d). Similarly, the expression of cyclin H, MAT1, CDK7, and p-CDK2 also decreased in cyclin H silenced ovarian cancer (Additional file 3: Fig. S3).

## Discussion

In the past decade, the incidence and the mortality rate of ovarian cancer in China have increased rapidly [16, 17]. Currently, the clinical treatment for ovarian cancer is surgery combined with chemotherapy drugs (cisplatin, carboplatin, etc.) [5, 18]. However, ovarian cancer is susceptible to chemotherapy resistance and prone to recurrence and metastasis after surgery [19, 20]. Ovarian cancer is a great challenge to gynecological oncologists, and it is urgent to identify new prognostic factors and develop new strategies in improving the outcome of ovarian cancer patients. In this study, we demonstrate the high expression of cyclin H in ovarian cancer and the association of cyclin H with the unfavorable clinicopathologic variables of patients.

The destruction of the cell cycle regulatory mechanism leads to uncontrolled cell growth. The cell cycle is a complicated and delicate process that is strictly regulated by the activation and inactivation of cyclin, CDK, and CDKIs. Cyclin and CDK are regulatory and catalytic subunits that play key roles in regulating cell cycle initiation and phase transition. Each of the Cyclins and the CDKs are expressed in a specific phase of the cell cycle where they control the progress of that phase and then switch onto the next phase. Cyclin H is a polypeptide consisting of 323 amino acids, forming CAK with CDK7 and MAT1 [21]. CAK forms the core part of TFIIH, which phosphorylates the CTD subunit of RNA polymerase II (RNAPII) and then participates in the transcription [22, 23]. CAK also phosphorylates CDK2 and promotes the cell cycle from G1 to S phase [24].

Emerging evidence has indicated that abnormal expression or genetic polymorphism of cyclin H is associated with tumor progression and chemosensitivity [13, 25, 26]. Here, we observed that cyclin H was positively correlated with the increased tumor grade of ovarian cancer and the expression of the proliferation markers Ki-67 and p-CDK2. Similar results are reported by diffuse large B-cell lymphoma [27], esophageal squamous cell carcinoma [12], and breast cancer [11]. Prashant Bavi et al.’s study concluded that a reduced or an absent cyclin H expression was significantly associated with the poor overall survival of patients with diffuse large B-cell lymphoma [27]. Hetal Patel and colleagues also described that Cyclin H expression was associated with a better patient outcome in breast cancer [11]. However, with our protein and mRNA data, we arrived at an opposite conclusion in the case of ovarian cancer, where the five-year survival rate in cyclin H ^low^ patients was significantly higher than in the cyclin H ^high^ patients. Consistent with our research, cyclin H positivity was significantly associated with the reduced disease-specific survival in patients with gastrointestinal stromal tumors [14]. These findings suggest that cyclin H may play different roles in different types of tumors.

The transition of G1 to S phase is a key step in the cell cycle process and plays an important role in developing most of the tumors. Suppressing G1/S transition may provide an attractive therapeutic target to stop cancer cells from proliferating [28]. Previously, a study reported that the down-regulation of cyclin H-CDK7 was implicated in the arrest of liver cancer cells in the G1 phase [29]. To further investigate the role of cyclin H in ovarian cancer cells, cyclin H in HO8910 cells was knocked down, and the cell cycle was detected after synchronizing the cell cycle by serum starvation and then serum release. We found that cyclin H shRNA resulted in G1/S cell cycle arrest and inhibited the proliferation of HO8910 cells. The expression of cyclin H, along with the associated cofactors CDK7 and MAT1, gradually increased after serum stimulation. The phosphorylation of CDK2 also showed a similar trend with CAK components, suggesting that cyclin H may promote G1/S transition by enhancing the phosphorylation of CDK2. Animal experiments confirmed that cyclin H has a positive regulatory role in ovarian cancer, and the knockdown of cyclin H suppresses the growth of tumors in nude mice. The expression of CDK7 and MAT1 along with the phosphorylation of CDK2 was decreased in cyclin H silenced xenografts.

## Conclusion

These findings indicate that high expression of cyclin H in ovarian cancer is associated with the poor prognosis of patients and promotes tumor growth through cell cycle regulation. In the past decades, tremendous efforts were put into developing inhibitors of CDKs, and many inhibitors were described as well, but most of them failed rigorous clinical testing [30]. To minimize the impact on normal proliferating cells, therapies that target cell cycle progression are always used in combination [31]. This study reveals a candidate that helps in early diagnosis or predicts prognosis of ovarian cancer and also provides evidence to support the therapeutic value of cyclin H in ovarian cancer, which may help in improving the diagnosis and the prognostic classification of ovarian cancers. Considering the reports of the controversial role of cyclin H in different kinds of tumors, more in-depth, systematic studies are needed to confirm the therapeutic value of cyclin H in ovarian cancer.

## Supplementary information

**Additional file 1: Figure S1.** Expression of cyclin H in different ovarian cancer cell lines. The protein levels of cyclin H in HO8910, SK-OV-3, and NIH: OVCAR-3 cells were detected by western blot. Cyclin H was highly expressed in HO8910 cells.

**Additional file 2: Figure S2.** Effect of cyclin H in migration and invasion of ovarian cancer cells. **(A)** Cell scratch test was used to evaluate the migration ability of HO8910 cells. HO8910 cells were seeded in a six-well plate at a density of 5 × 10^5^/well, and a straight scratch was made using a (yellow) pipette tip when the cultures are confluent. Twelve hours later, the distance of the wound was analyzed and the healing percentage was calculated. Bar = 200 µm. (B) Invasion of HO8910 cells was measured by transwell invasion assay. Upper chamber of 24-well transwell was blocked with Matrigel, and HO8910 cells were placed. After incubation for 20 h, the non-invaded cells on the top of the transwell were removed with a cotton swab and the invaded cells were counted under a light microscope. Bar = 20 µm.

**Additional file 3: Figure S3.** Body weight of nude mice after tumor inoculation. The body weight of mice was compared with that observed on day 0, and no significant change was found between the cyclin H shRNA group and the control group.

## Data Availability

Not applicable.

## Abbreviations

CAK: CDK-activating kinase
CDK: Cyclin-dependent kinase
CDKI: Cyclin-dependent kinase inhibitor
MAT1: for ménage à trois 1
RNAPII: RNA polymerase I
FBS: fetal bovine serum
