# Supplementary figures and images for: Cyclin H predicts the poor prognosis and promotes the proliferation of ovarian cancer
Source: Cancer Cell Int. 2020 Jul 16;20:316. doi: 10.1186/s12935-020-01406-5 (PMC7364476; doi:10.1186/s12935-020-01406-5)

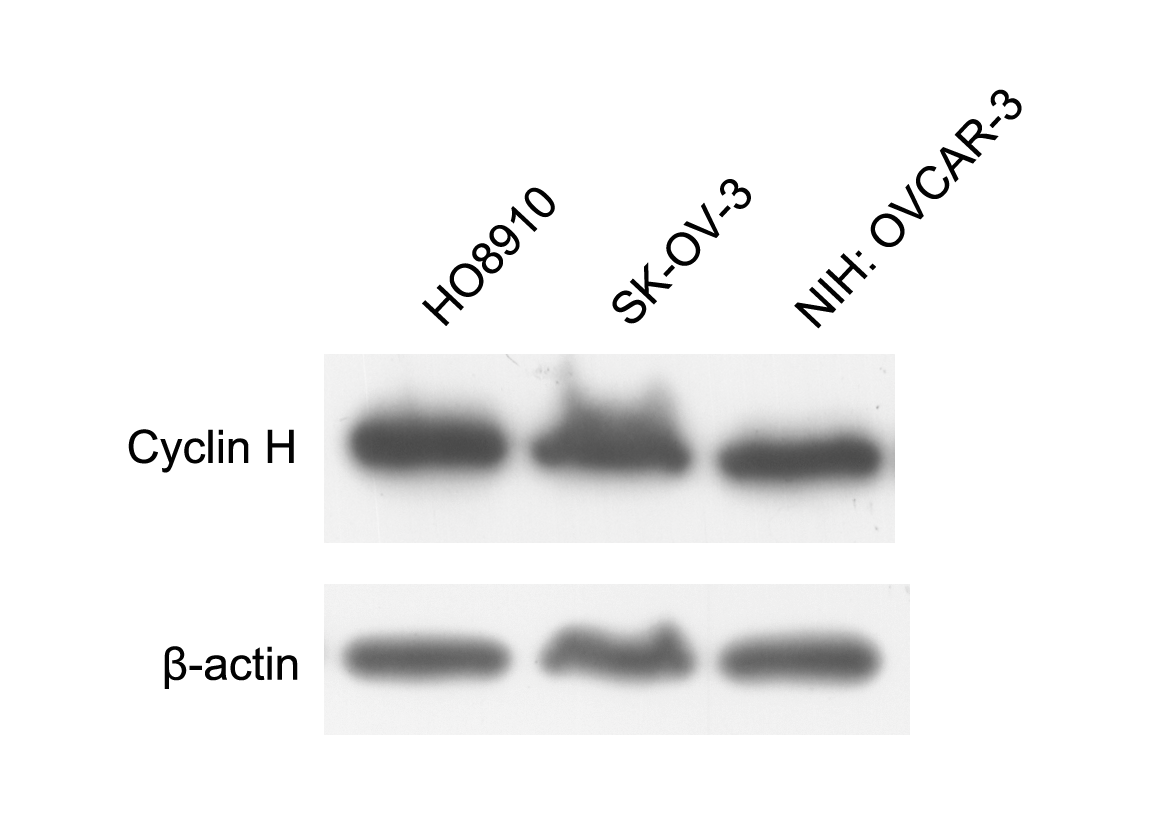

Supplement: Supplementary file 1 — Additional file 1: Figure S1. Expression of cyclin H in different ovarian cancer cell lines. The protein levels of cyclin H in HO8910, SK-OV-3, and NIH: OVCAR-3 cells were detected by western blot. Cyclin H was highly expressed in HO8910 cells. [file 12935_2020_1406_MOESM1_ESM.tif]

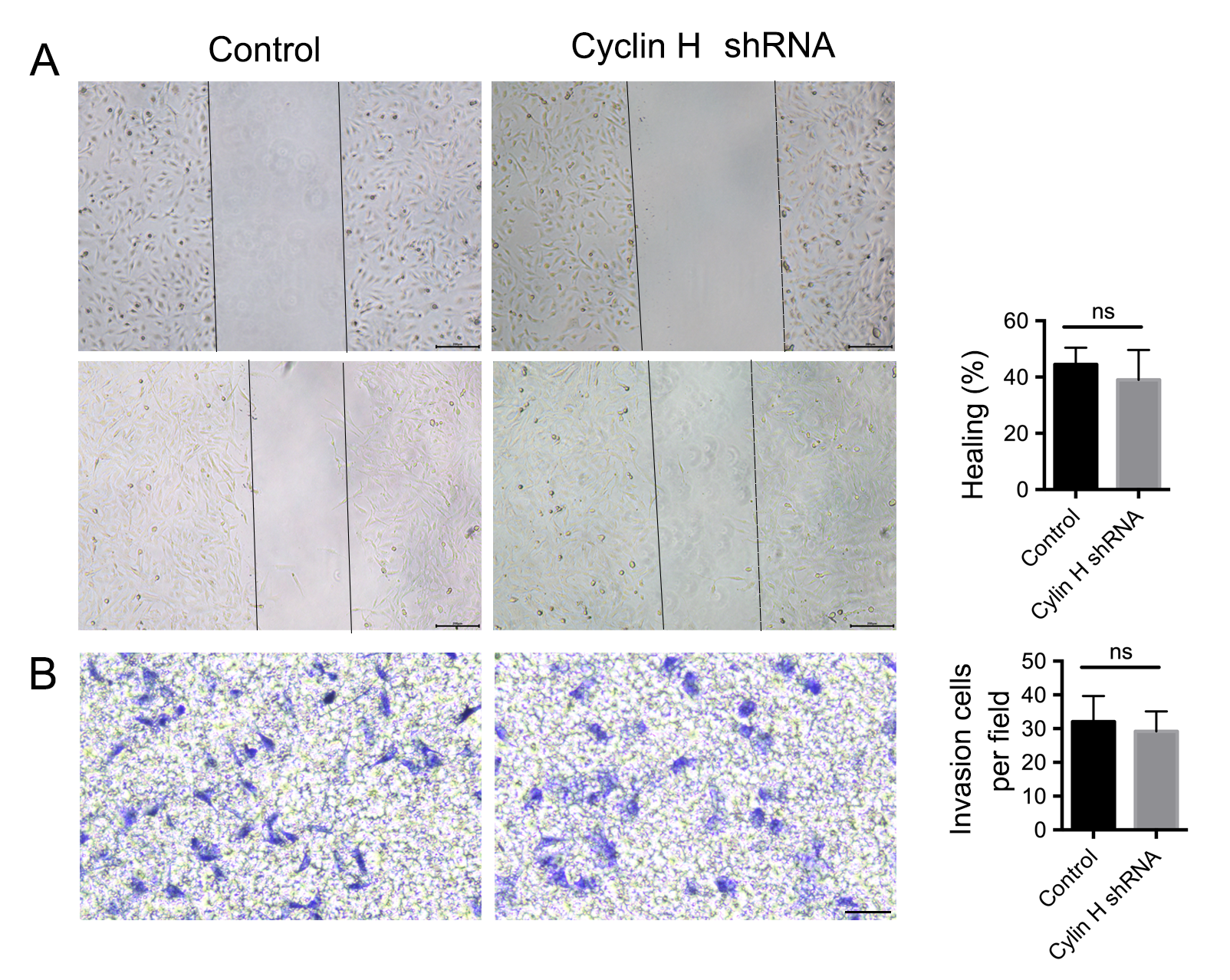

Supplement: Supplementary file 2 — Additional file 2: Figure S2. Effect of cyclin H in migration and invasion of ovarian cancer cells. (A) Cell scratch test was used to evaluate the migration ability of HO8910 cells. HO8910 cells were seeded in a six-well plate at a density of 5 × 10^5^/well, and a straight scratch was made using a (yellow) pipette tip when the cultures are confluent. Twelve hours later, the distance of the wound was analyzed and the healing percentage was calculated. Bar = 200 µm. (B) Invasion of HO8910 cells was measured by transwell invasion assay. Upper chamber of 24-well transwell was blocked with Matrigel, and HO8910 cells were placed. After incubation for 20 h, the non-invaded cells on the top of the transwell were removed with a cotton swab and the invaded cells were counted under a light microscope. Bar = 20 µm. [file 12935_2020_1406_MOESM2_ESM.tif]

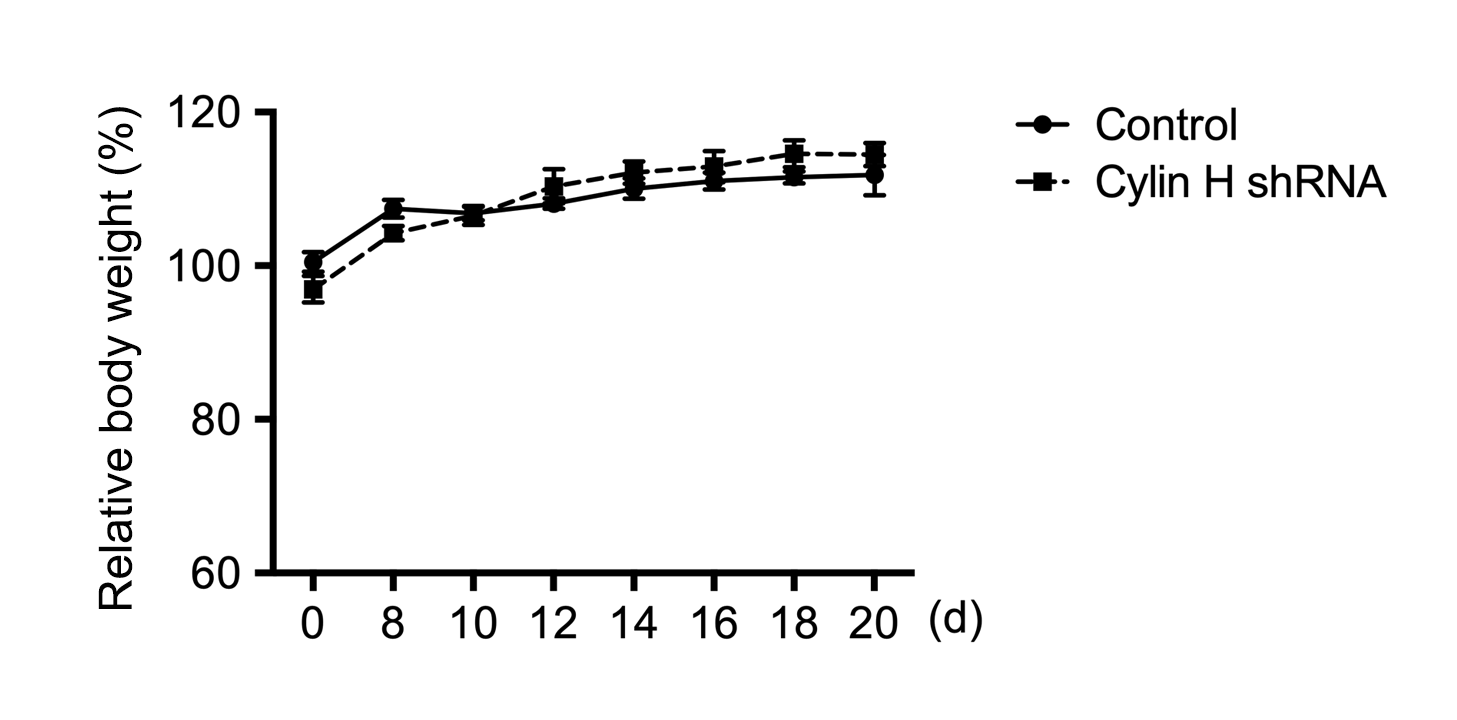

Supplement: Supplementary file 3 — Additional file 3: Figure S3. Body weight of nude mice after tumor inoculation. The body weight of mice was compared with that observed on day 0, and no significant change was found between the cyclin H shRNA group and the control group. [file 12935_2020_1406_MOESM3_ESM.tif]
